# Proactive classical biological control of *Lycorma delicatula* (Hemiptera: Fulgoridae) in California (U.S.): Host range testing of *Anastatus orientalis* (Hymenoptera: Eupelmidae)

**DOI:** 10.3389/finsc.2023.1134889

**Published:** 2023-02-20

**Authors:** Francesc Gómez Marco, Douglas Yanega, Marta Ruiz, Mark S. Hoddle

**Affiliations:** ^1^ Department of Entomology, University of California, Riverside, CA, United States; ^2^ Department of Botany & Plant Sciences, University of California, Riverside, CA, United States; ^3^ Center for Invasive Species Research, University of California, Riverside, CA, United States

**Keywords:** Coreidae, Lasiocampidae, Spotted lanternfly, Saturniidae, parasitoid, polyphagy, southwestern U.S

## Abstract

*Lycorma delicatula* (Hemiptera: Fulgoridae), the spotted lanternfly, native to China, invaded and established in the northeast U.S. in 2014. Since this time, populations have grown and spread rapidly, and invasion bridgeheads have been detected in mid-western states (i.e., Indiana in 2021). This invasive pest presents a significant threat to Californian agriculture. Therefore, a proactive classical biological control program using *Anastatus orientalis* (Hymenoptera: Eupelmidae), a *L. delicatula* egg parasitoid native to China, was initiated in anticipation of eventual establishment of *L. delicatula* in California. In support of this proactive approach, the potential host range of *A. orientalis* was investigated. Eggs of 34 insect species either native or non-native to the southwestern U.S. were assessed for suitability for parasitism and development of *A. orientalis*. Of the native species tested, 10, 13, and one were Hemiptera, Lepidoptera, and Mantodea, respectively. Of the non-native species, eight Hemiptera and two Lepidoptera were evaluated. Host range tests conducted in a quarantine facility, exposed individually mated *A. orientalis* females (Haplotype C) to non-target and target (i.e., *L. delicatula*) eggs in sequential no-choice and static choice experiments to determine suitability for parasitization and development. Additionally, the sex ratio, fertility, and size of offspring obtained from non-target and target eggs were evaluated. Results of host range testing indicated that *A. orientalis* is likely polyphagous and can successfully parasitize and develop in host species belonging to at least two different orders (i.e., Hemiptera, Lepidoptera) and seven families (Coreidae, Erebidae, Fulgoridae, Lasiocampidae, Pentatomidae, Saturniidae and Sphingidae). Prospects for use of *A. orientalis* as a classical biological control agent of *L. delicatula* in the southwestern U.S. are discussed.

## 1 Introduction

Classical or introduction biological control is the intentional importation, release, and establishment of natural enemies for suppressing damaging populations of invasive non-native organisms to densities that no longer cause economic or ecological harm. This approach aims to reduce pest population densities by re-associating safe (i.e., host-specific) and efficacious natural enemies with the target pest (1). Host range and host specificity testing are important primary steps in identifying natural enemy species that may have deleterious impacts on pest populations while presenting minimal risk to non-target species (1). Host use evaluation studies are mandatory in the United States of America (U.S.) and provide safety data for review by Federal agencies (i.e., United States Department of Agriculture, Animal and Plant Health Inspection Service [USDA APHIS]), that regulate the importation and release of natural enemies for use in classical biological control programs (2). Host range and host specificity testing evaluations are time consuming, often taking years to complete (3). During this time, newly established invasive pest populations tend to increase in density and spread as management plans are slowly developed and implemented. Proactive biological control attempts to reduce or eliminate this window of opportunity for an invasive pest by evaluating candidate natural enemies for potential use in a classical biological control program in advance of the anticipated incursion and establishment of the target pest in the area of concern (3).

Spotted lanternfly, *Lycorma delicatula* (White) (Hemiptera: Fulgoridae), native to China (4), was detected for the first time in the U.S. in Berks County Pennsylvania, in September 2014 (5). By November 2022, *L. delicatula* infestations were confirmed in an additional 13 states in eastern and mid-western areas of the U.S. (Connecticut, Delaware, Indiana, Maryland, Massachusetts, Michigan, New Jersey, New York, North Carolina, Ohio, Rhode Island, Virginia, and West Virginia) (6). *Lycorma delicatula* is a phloem-feeding fulgorid that has a broad host range (7). Direct feeding damage can cause mortality to highly preferred hosts like *Ailanthus altissima* (Miller) (Sapindales: Simaroubaceae) and grapevines (*Vitis vinifera* L. [Vitales: Vitaceae]). Indirect damage results from the excretion of high quantities of honeydew that promote sooty mold growth (7, 8). *Lycorma delicatula* has been recorded infesting forest and ornamental shade tree species in natural and urban areas, respectively (7, 9), and presents a significant risk to economically important perennial agricultural crops (e.g., grapes and nuts) (10, 11). Long-distance dispersal by *L. delicatula* is almost entirely human-assisted. This occurs primarily through the accidental translocation of egg masses that are often laid indiscriminately on inert substrates (e.g., wooden pallets and railcars) that undergo subsequent transportation into uninfested areas (12, 13). This type of inadvertent relocation in the U.S. likely resulted in the establishment of invasion bridgeheads in Indiana (2021) and Michigan and North Carolina (2022) (6). Ecological niche models indicate that *L. delicatula* has a potential distribution that includes large areas of the west coast of the U.S., and other parts of the world (e.g., Europe) (14). For California, a western U.S. state, with an agricultural economy worth ~$50 billion per year (15), *L. delicatula* is viewed as a significant invasion threat that could cause major problems for producers of specialty crops, like grapes and nuts, which are multi-billion-dollar industries (15).

The egg parasitoid, *Anastatus orientalis* Yang and Choi (Hymenoptera: Eupelmidae), was found parasitizing *L. delicatula* eggs in northern China in 2011during foreign exploration for natural enemies for use in South Korea, where *L. delicatula* is also invasive (16, 17). Following the invasion and spread of *L. delicatula* on the east coast of the U.S. there was renewed interest in the potential use of *A. orientalis* as a classical biological control agent (18, 19). Molecular analyses identified six different haplotypes of *A. orientalis* collected from the native range. Importations of *A. orientalis* into the U.S. were initially comprised of Haplotype C, which was first evaluated as a classical biological control agent against *L. delicatula* (20). The majority of *Anastatus* spp. Motschulsky are primary endoparasitoids attacking eggs of Diptera, Dictyoptera, Coleoptera, Hemiptera, Lepidoptera, Orthoptera, and Mantodea (21–27). Numerous *Anastatus* species have been considered or used as classical biological control agents against various pests around the world. For example, *A. japonicus* Ashmead was released in the eastern U.S. for control of *Lymantria dispar* L. (Lepidoptera: Erebidae) (27, 28) and against *Tessaratoma papillosa* Drury (Hemiptera: Pentatomidae) in China (29, 30). *Anastatus* sp. was released in Nepal to control *Rhynchocoris humeralis* (Thunberg) (Hemiptera: Pentatomidae) (31). *Anastatus bifasciatus* (Geoffroy) was evaluated to study levels of biotic resistance of central European natural enemies against invasive *Halyomorpha halys* Stål (Hemiptera: Pentatomidae) populations (32, 33). In the eastern U.S., *A. reduvii*, a native species, has been detected as one of the most common parasitoids emerging from eggs of invasive *H. halys* (34). Some *Anastatus* spp. are commercially-available and used for augmentative biological control of *Amblypelta nitida* Stål and *A. lutescens lutescens* Distant (Hemiptera: Coreidae) in Australia (35, 36).

Given the obvious threat posed to California agriculture by the westward migration of *L. delicatula* in the U.S., a proactive biological control program was initiated. Proactive research efforts focused on the suitability of *A. orientalis* (Haplotype C) as a potential classical biological control agent of *L. delicatula* in advance of its expected establishment in California (3). Consequently, the objective of this study was to investigate the physiological host range of *A. orientalis* on native and non-native non-target species from the southwestern U.S. (i.e., California and Arizona) to determine whether or not this natural enemy would be a suitable candidate to release for classical biological control of *L. delicatula* should it eventually establish in California. The results of these studies are presented here.

## 2 Materials and methods

### 2.1 Source and collection of test insects

*Anastatus orientalis* Haplotype C specimens were obtained from colonies established at USDA APHIS PPQ (Plant Protection and Quarantine) Forest Pest Methods Laboratory in Massachusetts, U.S. Initial *A. orientalis* populations were shipped to the University of California Riverside Insectary and Quarantine Facility (UCR-IQF) as parasitized *L. delicatula* egg masses under USDA-APHIS permit P526P-22-03022 and P526P-22-04208 and California Department of Food and Agriculture (CDFA) permit 3888. Colonies of *A. orientalis* were established in UCR-IQF in October 2019 and reared continuously on cold stored *L. delicatula* egg masses.

*Lycorma delicatula* egg masses were field collected in winter (December to March) of 2019 to 2022. Collections were made in Pennsylvania, U.S. (Berks, Dauphin, Huntingdon, Lancaster and Lebanon Counties) predominantly from *A. altissima* (>90%). Entire egg masses attached to underlying bark were removed using chisels and shipped or hand carried to the UCR-IQF under USDA-APHIS permit P526P-19-02058 and CDFA Permit 3458. In quarantine, all field collected egg masses were stored at 5°C and 60-75% R.H. Egg masses were randomly selected and used for experiments reported here.

Selection of non-target species for host range testing was made based on phylogenetic relationships amongst species within the family Fulgoridae and their representation in the southwestern U.S. Five native fulgorid genera in California and Arizona, *Amcyle* spp., *Cyrpoptus* spp., *Poblicia* spp., *Scaralina* spp. (described incorrectly as genus *Alphina* spp Stål, Yanega et al. unpublished), and *Scolopsella* spp (37). were targeted for field collections and use in host range tests. Previous host range studies on *Anastatus* suggested that species may potentially have broad host ranges and are capable of utilizing hosts from different orders (32). Consequently, to determine if *A. orientalis* potentially exhibits oligophagy or polyphagy, additional non-target species belonging to Hemiptera (Cicadellidae, Coreidae, Liviidae, Pentatomidae, Reduviidae, and Rhopalidae), Lepidoptera (Erebidae, Lasiocampidae, Saturniidae, and Sphingidae), and Mantodea (Mantidae) were included in host range testing for *A. orientalis* (**Table 1**). These families were also selected to compliment simultaneous testing conducted by collaborators at the USDA APHIS PPQ Forest Pest Methods Laboratory of potential native and non-native non-target species found in the eastern U.S. All non-target insect colonies used for host range testing, unless otherwise stated, were maintained on each test species preferred host plant species held in cages (BugDorm-2120 61×61×61 cm, MegaView Science Co. Ltd., Taiwan) at the UCR-IQF at 25°C, 65%RH, L:D 16:8. Colonies were checked daily for egg masses which were harvested and used immediately or held at 10°C until used for experiments.

**Table 1:** Non-target species tested, selection criteria for use in evaluations, and collection information.

Order	Family	Genera	Species	Native, non-native or invasive	Egg deposition type^1^	Selection criteria (Ref)	GPS coordinates of collection sites^2^/Commercially obtained	Collection date
Hemiptera	Cicadellidae	*Homalodisca*	*vitripennis*	Invasive	M	Egg masses laid under leaf epidermis	33° 58’ 20.26”N - 117° 19’ 3.33”W (CA)	April 2020
	Coreidae	*Acanthocephala*	*thomasi*	Native	I	Recorded host family for *Anastatus* sp (29, 32).	31° 54’ 54.38”N - 109° 8’ 9.17”W (AZ)	August 2021
		*Chelinidea*	*vittiger*	Native	G	Recorded host family for *Anastatus* sp (29, 32).	33° 58’ 29.32”N - 117° 18’ 59.98”W (CA)	July 2020
		*Leptoglossus*	*zonatus*	Invasive	G	Recorded host family for *Anastatus* sp (29, 32).	Colonies maintained at UC Riverside	–
		*Thasus*	*neocalifornicus*	Native	G	Recorded host family for *Anastatus* sp (29, 32).	31° 40’ 42.93”N - 110° 39’ 37.16”W (AZ)	August 2021
	Fulgoridae	*Cyrpoptus*	*vanduzeei*	Native	M	Family-level relatedness to *L. delicatula*	31° 54’ 48.28”N - 109° 8’ 22.75”W (AZ)	August 2020
		*Lycorma*	*delicatula*	Invasive	M	Target	Pennsylvania, U.S.	2019, 2020 and 2021
		*Poblicia*	*fuliginosa*	Native	M	Family-level relatedness to *L. delicatula*	Southeastern AZ	August 2021
		*Scaralina*	*unidentified* spp.	Native	M	Family-level relatedness to *L. delicatula*	31° 53’ 12.77”N - 109° 12’ 40.37”W (AZ)	August 2019 to 2021
	Liviidae	*Diaphorina*	*citri*	Invasive	G	Eggs readily available from research colonies.	Colonies maintained at UC Riverside	–
	Pentatomidae	*Banasa*	*dimidiata*	Native	M	Recorded host family for *Anastatus* sp (29).	33° 58’ 20.26”N - 117° 19’ 3.34”W (CA)	April 2020
		*Bragada*	*hilaris*	Invasive	I	Recorded host family for *Anastatus* sp (29).	Colonies maintained at UC Riverside	–
		*Chinavia*	*hilaris*	Native	M	Recorded host family for *Anastatus* sp (29).	Colonies maintained at UC Riverside	–
		*Nezara*	*viridula*	Invasive	M	Recorded host species for *Anastatus* sp (21, 29).	34° 3’ 46.03”N - 118° 21’ 16.12”W (CA)	April 2020
		*Halyomorpha*	*halys*	Invasive	M	Recorded host species for *Anastatus* sp (29, 31).	34° 3’ 46.03”N - 118° 21’ 16.12”W (CA)	April 2020
	Reduviidae	*Zelus*	*renardii*	Non-native	M	Beneficial insect. Readily available	Commercially available	–
	Rhopalidae	*Jadera*	*haematoloma*	Invasive	I	Easily collected from field sites	33° 58’ 26.67”N - 117° 19’ 1.55”W (CA)	April 2020
Mantodea	Mantidae	*Stagmomantis*	*californica*	Native	M	Recorded host family for *Anastatus* sp (22, 23).	33° 40’ 12.00”N - 116° 24’ 44.43”W (CA)	August 2020
Lepidoptera	Erebidae	*Apantesis*	*unidentified* sp.	Native	I	Recorded host family for *Anastatus* sp (29).	33° 27’ 57.48”N - 117° 2’ 29.93”W (CA)	June 2020
		*Pseudohemihyalea*	*edwardsii*	Native	G	Recorded host family for *Anastatus* sp (29).	31° 53’ 12.77”N- 109° 12’ 40.37”W (AZ)	August 2019 to 2021
	Lasiocampidae	*Gloveria*	*arizonensis*	Native	G	Recorded host family for *Anastatus* sp (29).	31° 53’ 12.77”N- 109° 12’ 40.37”W (AZ)	August 2019 to 2021
	Saturniidae	*Actias*	*luna*	Non-native	I	Recorded host family for *Anastatus* sp (29).	Commercially available	–
		*Agapema*	*anona*	Native	I	Recorded host family for *Anastatus* sp (29).	31° 43’ 5.15”N - 110° 52’ 56.22”W (AZ)	September 2021
		*Anisota*	*oslari*	Native	G	Recorded host family for *Anastatus* sp (29).	31° 53’ 12.77”N- 109° 12’ 40.37”W (AZ)	August 2021
		*Antheraea*	*oculea*	Native	G	Recorded host genera for *A. orientalis* (38).	31° 53’ 12.77”N- 109° 12’ 40.37”W (AZ)	August 2021
		*Automeris*	*cecrops pamina*	Native	G	Recorded host family for *Anastatus* sp (29).	31° 53’ 12.77”N- 109° 12’ 40.37”W (AZ)	August 2021
			*metzli*	Non-native	G	Recorded host family for *Anastatus* sp (29).	Commercially available	–
		*Eupackardia*	*calleta*	Native	I	Recorded host family for *Anastatus* sp (29).	31° 43’ 5.15”N - 110° 52’ 56.22”W (AZ)	August 2021
		*Hyalophora*	*euryalus*	Native	I	Recorded host family for *Anastatus* sp (29).	32° 54’ 55.26”N - 116° 53’ 50.67”W (CA)	May 2021
		*Rothschildia*	*cincta*	Native	I	Recorded host family for *Anastatus* sp (29).	31° 43’ 5.15”N - 110° 52’ 56.22”W (AZ)	August 2021
		*Saturnia*	*walterorum*	Native	I	Recorded host family for *Anastatus* sp (29).	32° 54’ 55.26”N - 116° 53’ 50.67”W (CA)	May 2021
	Sphingidae	*Pachysphinx*	*occidentalis*	Native	I	Recorded host family for *Anastatus* sp (29).	31° 53’ 12.77”N- 109° 12’ 40.37”W (AZ)	August 2025

(1) Egg type: M = Egg mass [eggs laid very close together with some protective material covering the eggs]; I = Individual eggs; G = Group of individual eggs laid in patches of irregular number of eggs.

(2) CA, California; AZ, Arizona. California and Arizona are southwestern states in the U.S.

(Ref), Reference.

#### 2.2.1 Hemiptera

##### 2.2.1.1 Fulgoridae collections

**Figure 1:**
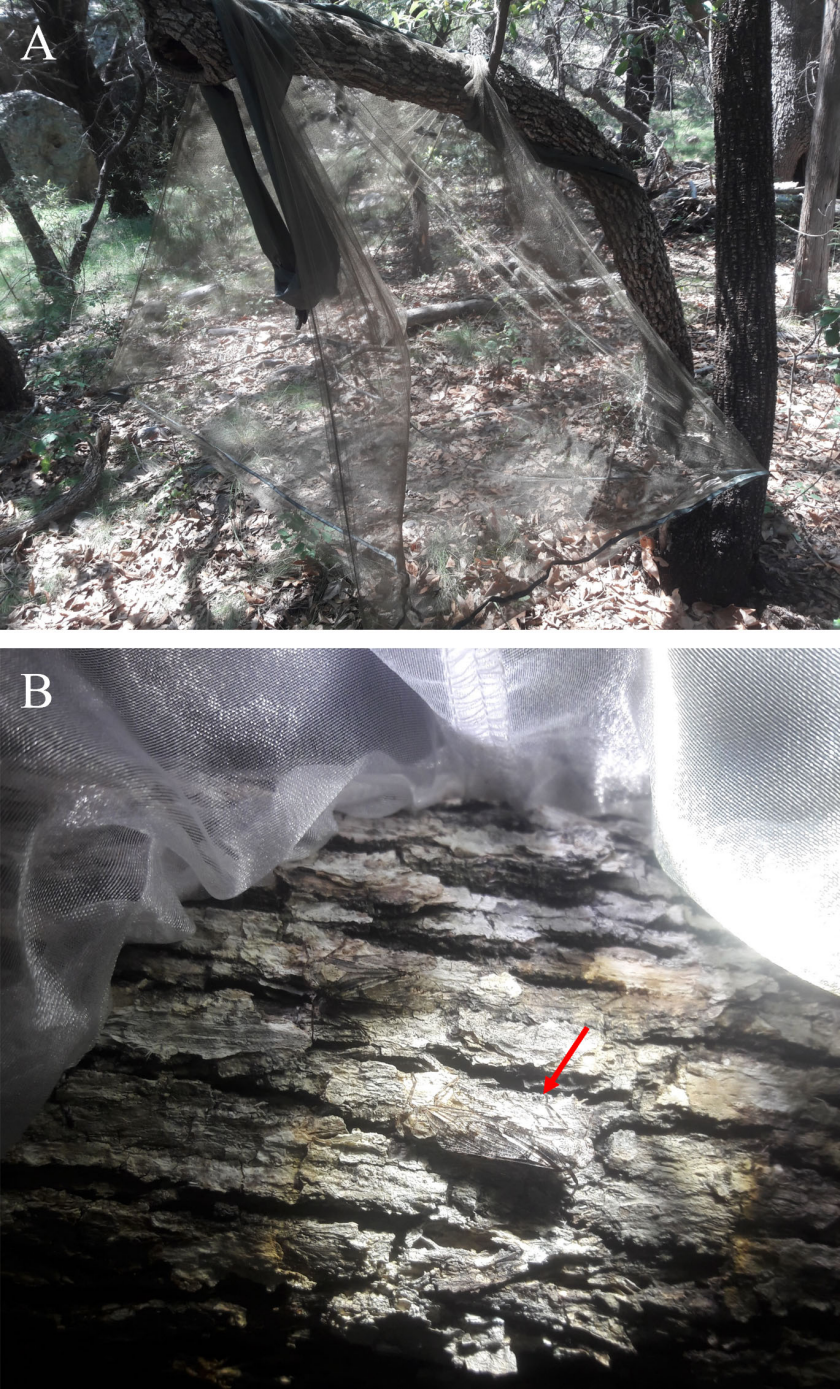
**(A)** Sleeve cages set up on branches of *Quercus arizonica* in Portal, AZ were used to confine adult native fulgorids on putative host plants for mating and oviposition. **(B)** A *Scaralina* sp. specimen captured at night by black lighting is seen resting on the bark of *Q. arizonica* branch enclosed by a sleeve cage (the red arrow indicates position of *Scaralina* sp.).

Fulgorids collected in the Chiricahua mountains near Portal, Santa Cruz County, in southeastern Arizona included *Scaralina* spp. (comprised of three undescribed species and incorrectly placed as *Alphina* genera, Yanega et al. unpublished) and *Cyrpoptus vanduzeei* Ball (**Table 1**). Adult *Scaralina* spp. were hand collected as they were attracted to mercury vapor and UV lights. Immediately after capture, adult males and females were caged (i.e., sleeve cages made of mesh with fiber spacing of 160 µm (**Figure 1**) on trunks of oak trees, *Quercus arizonica* Sarg. (Fagales: Fagaceae), at the American Museum of Natural History’s Southwestern Research Station, Portal Arizona. Cages were inspected daily for oviposited egg masses which were collected and maintained at ~10°C until use in experiments with *A. orientalis*. Three species of *Scaralina* were collected and relatively low numbers of egg masses per species were obtained. Therefore, all egg masses (n = 9) used for experiments were pooled and referred to as “*Scaralina* spp.”. *Poblicia fuliginosa* (Olivier) and *C. vanduzeei* adults were collected during the day from *Baccharis sarothroides* Gray (Asterales: Asteraceae) from different locations in Arizona (**Table 1**). Adult *P. fuliginosa* and *C. vanduzeei* were maintained on potted *B. sarothroides* plants held in cages. Live insects used for experiments were moved to UCR-IQF under USDA-APHIS Permit number P526P-19-00766 and CDFA Permit number 3457.

##### 2.2.1.2 Cicadellidae collections

*Homalodisca vitripennis* Germar (Hemiptera: Cicadellidae), a pest of grapes, were collected with sweep nets in citrus orchards in Riverside, California and maintained on potted basil, *Ocimum basilicum* (Lamiales: Lamiaceae), a host plant that supports adult feeding and oviposition.

##### 2.2.1.3 Coreidae collections

*Acanthocephala thomasi* Uhler, *Chelinidea vittiger* Uhler, *Leptoglossus zonatus* (Dallas) and *Thasus neocalifornicus* Brailovsky and Barrera were included in host range tests. *Acanthocephala thomasi* specimens were hand collected in Portal Arizona (**Table 1**) and maintained on potted *B. sarothroides* plants. *Chelinidea vittiger* specimens were collected in Riverside, California and maintained on *Opuntia* sp. (Caryophyllales: Cactaceae). *Leptoglossus zonatus* specimens were obtained from research colonies maintained in the Department of Entomology at UC Riverside. *Thasus neocalifornicus* were collected in Sonoita, Arizona and maintained on *Prosopis velutina* Wooton (Fabales: Fabaceae) trees (**Table 1**).

##### 2.2.1.4 Liviidae collections

*Diaphorina citri* Kuwayama (Hemiptera: Liviidae) eggs were obtained from colonies maintained in the UCR-IQF building of the Department of Entomology at UCR. *Diaphorina citri*, collected in southern California in 2011 and certified free of the citrus killing bacterium *Candidatus* Liberibacter asiaticus (*C*Las), were reared on *Murraya koenigii* (Sapindales: Rutaceae), a non-propagative host for *C*Las.

##### 2.2.1.5 Pentatomidae collections

*Banasa dimidiata* (Say) (native), *Bragada hilaris* (Burmeister) (invasive), *Chinavia hilaris* Say (native), *Nezara viridula* L. (invasive) and *H. halys* (invasive) were used in host range tests. *Banasa dimidiata* specimens were collected in Riverside, California and maintained on *Hirschfeldia incana* (Brassicales: Brassicaceae) (**Table 1**). *Bragada hilaris* and *Chinavia hilaris* were obtained from research colonies maintained in the Department of Entomology at UCR. *Halyomorpha halys* and *N. viridula* were established from adults collected from Hancock Park, in Los Angeles, California. All live insects were transported to UCR-IQF under USDA-APHIS Permit number P526P-22-03011 and CDFA Permit number 3887 (**Table 1**). *Halyomorpha halys* colonies were maintained on a mixed diet of avocados, carrots, apples, green beans, table grapes and *A. altissima. Nezara viridula* colonies were maintained on green bean plants and raw peanuts.

##### 2.2.1.6 Reduviidae collections

Egg masses of *Zelus renardii* Kolenati were purchased from Arbico Organics (Oro Valley, Arizona). Purchased eggs were approximately 2 days of age upon receipt. Egg masses were exposed to *A. orientalis* immediately.

##### 2.2.1.7 Rhopalidae collections

*Jadera haematoloma* Herrich-Schäffer adults were collected in the Botanic Gardens at the University of California Riverside campus, Riverside, California. Specimens were not feed, adults were kept on ventilated plastic containers, provided with a water-saturated cotton wick, and eggs were collected daily and exposed immediately to *A. orientalis*.

#### 2.2.2 Lepidoptera

All Lepidoptera (Erebidae, Lasiocampidae, Saturniidae, and Sphingidae) species, used in host range testing (except for *Automeris metzli* Sallé which were purchased from an online vendor as pupae) were field collected as adults (**Table 1**). *Automeris metzli* pupae were held at 10°C for two months to simulate exposure to winter temperatures. After this chilling period, pupae were moved to a temperature cabinet set at 25 ± 2°C and 60% R.H. until adults emerged. Field captured adult moths were kept in bug-dorms (BugDorm-2120 61×61×61 cm, MegaView Science Co. Ltd., Taiwan) and maintained outdoors near collection sites or in a temperature chamber (25 ± 2°C; 60% of R.H.) when moved into UCR-IQF for mating and oviposition. Adults were not provided with food or water as test species do not feed in adult stage (except *Apantesis* sp. which was provided 50% honey water solution). Eggs oviposited onto walls of cages or on cardboard oviposition strips were collected daily and either used immediately or maintained at 10°C until used for experiments.

#### 2.2.3 Mantodea

##### 2.2.3.1 Mantidae collections

Adult female *Stagmomantis californica* Rehn & Hebard were collected in Riverside County, California (**Table 1**) and fed with *H. halys* nymphs and adults. Ootheca, ~48 h of age, were collected and presented to *A. orientalis*.

### 2.3 No-choice sequential host-testing

Five female *A. orientalis*, less than 24 hours of age, were placed in a test unit with one male and a thin smear of pure honey on the mesh of the unit’s lid as a carbohydrate source. Each experimental egg mass-parasitoid test arena was comprised of a clear plastic container 3 cm × 4 cm × 5 cm (180 mL clear RPTE hinged lid deli containers, AD16 GenPak, Charlotte, NC) with a modified lid that had a ventilated mesh window (1.5 cm x 2.5 cm) to facilitate air exchange. One *L. delicatula* egg mass was placed in the test unit and exposed to the five females and the male of *A. orientalis* for seven days. This seven day period is a pre-oviposition period during which host feeding occurs (pers. obs. F. Gomez Marco) at temperatures that simulate the fall (i.e., September when parasitoid oviposition in the field occurs) in Beijing (average daily high 25°C, average daily low 14°C, lights on 6:00 AM, lights off 6:30 PM [i.e., L:D 12.5:11.5], 75% R.H.; referred to as Beijing-fall regimen), the general area where *A. orientalis* was collected for colony establishment in the U.S (16–19). All experiments were conducted under the Beijing-fall regimen. After this one-week pre-oviposition period, females were moved individually and placed singly without males in new separate test units for a total of 274 A*. orientalis* females. One non-target host egg mass [number of eggs in the egg mass and the physical size of the egg mass varied on species being tested (**Table 2**)] was placed into each test unit containing a single mated female for seven days. After the seven-day exposure period, non-target eggs were removed, and replaced with *L. delicatula* egg masses, and females were left to host feed and oviposit for an additional seven days. Thus, the sequential non-choice tests were performed in this order; target host (pre-oviposition period, seven days) – non-target host (seven days) – target host (seven days). Parasitism of *L. delicatula* egg masses in the exposure trial following exposure to non-target eggs confirmed female competency if no parasitism was observed from non-target exposures. The total time taken to complete each no choice sequential host test cycle for each female was 21 days.

**Table 2:** Non-target species tested for host range suitability of *A. orientalis*.

				7 days exposure to *A. orientalis*				24 hours exposure to *A. orientalis*			
Order	Family	Genera	Species	Percent parasitism (± SE)	(n)	Average number of eggs/n (± SE)	Sex ratio (± SE)	Percent parasitism (± SE)	(n)	Average number of eggs/n (± SE)	Sex ratio (± SE)
Hemiptera	Cicadellidae	*Homalodisca*	*vitripennis*	0	5	10 ± 0.32	–	–	–	–	–
	Coreidae	*Acanthocephala*	*thomasi*	100	1	1	1	–	–	–	–
		*Chelinidea*	vittiger *	0	1	37	–	–	–	–	–
		*Leptoglossus*	*zonatus*	0	11	38.27 ± 2.67	–	–	–	–	–
		*Thasus*	*neocalifornicus*	0	7	4.71 ± 0.75	–	–	–	–	–
	Fulgoridae	*Cyrpoptus*	vanduzeei *	0	2	54.5	–	–	–	–	–
		*Lycorma*	delicatula **	58.24 ± 10.09	13	39.08 ± 3.84	0.77 ± 0.07	28.15 ± 5.04	38	40.66 ± 3.79	0.76 ± 0.04
		*Poblicia*	*fuliginosa*	12.37 ± 8.42	7	29.86 ± 2.69	0	–	–	–	–
		*Scaralina*	*spp.*	0	7	26.57 ± 2.57	–	–	–	–	–
	Liviidae	*Diaphorina*	*citri*	0	3	115.67 ± 40.71	–	–	–	–	–
	Pentatomidae	*Banasa*	*dimidiata*	0	1	14	–	–	–	–	–
		*Bragada*	*hilaris*	0	3	6 ± 0.58	–	–	–	–	–
		*Chinavia*	*hilaris*	68.79 ± 12.41	11	17.73 ± 2.51	0.22 ± 0.1	7.41	2	30.5	0
		*Nezara*	*viridula*	18.34 ± 6.87	17^a^	77.35 ± 5.79	0.07 ± 0.05	4.79 ± 1.91	17	59.58 ± 3.78	0.02 ± 0.01
		*Halyomorpha*	*halys*	48.05 ± 3.5	92^a^	26.85 ± 0.57	0.05 ± 0.02	37.27 ± 13.96	8	23 ± 2.46	0
	Reduviidae	*Zelus*	*renardii*	0	4	24.25 ± 2.02	–	–	–	–	–
	Rhopalidae	*Jadera*	*haematoloma*	0	3	43 ± 3.21	–	–	–	–	–
Mantodea	Mantidae	*Stagmomantis*	*californica*	0	2	150	–	–	–	–	–
Lepidoptera	Erebidae	*Apantesis*	*sp.*	0	2	130 ± 8	–	–	–	–	–
		*Pseudohemihyalea*	*edwardsii*	16.7 ± 6	6	22.17 ± 3.12	0	–	–	–	–
	Lasiocampidae	*Gloveria*	*arizonensis*	38.74 ± 9.62	13^a^	18.92 ± 1.72	0.11 ± 0.06	23.33 ± 9.55	5	16 ± 1	0.72 ± 0.21
	Saturniidae	*Actias*	*luna*	25.78 ± 6.89	9	19.11 ± 0.79	0.04 ± 0.04	–	–	–	–
		*Agapema*	*anona*	76.48 ± 14.10	5	42.2 ± 7.43	0	–	–	–	–
		*Anisota*	oslari *	0	16	14.63 ± 1.22	–	–	–	–	–
		*Antheraea*	*oculea*	0	1	6	–	–	–	–	–
		*Automeris*	*cecrops pamina*	19.12 ± 14.41	3	13 ± 3	0	–	–	–	–
			*metzli*	0	1	10	–	–	–	–	–
		*Eupackardia*	*calleta*	100	1	12	0	–	–	–	–
		*Hyalophora*	*euryalus*	100	3	5.5	0.16	–	–	–	–
		*Rothschildia*	*cincta*	45	2	10	0	–	–	–	–
		*Saturnia*	*walterorum*	19.60 ± 17.41	5	11.2 ± 1.02	0.5 ± 0.32	42.96 ± 25.7	3	9.67 ± 0.33	0.5 ± 0.2
	Sphingidae	*Pachysphinx*	*occidentalis*	1.56 ± 1.56	4	17.5 ± 2.72	0	–	–	–	–

**Figure 2:**
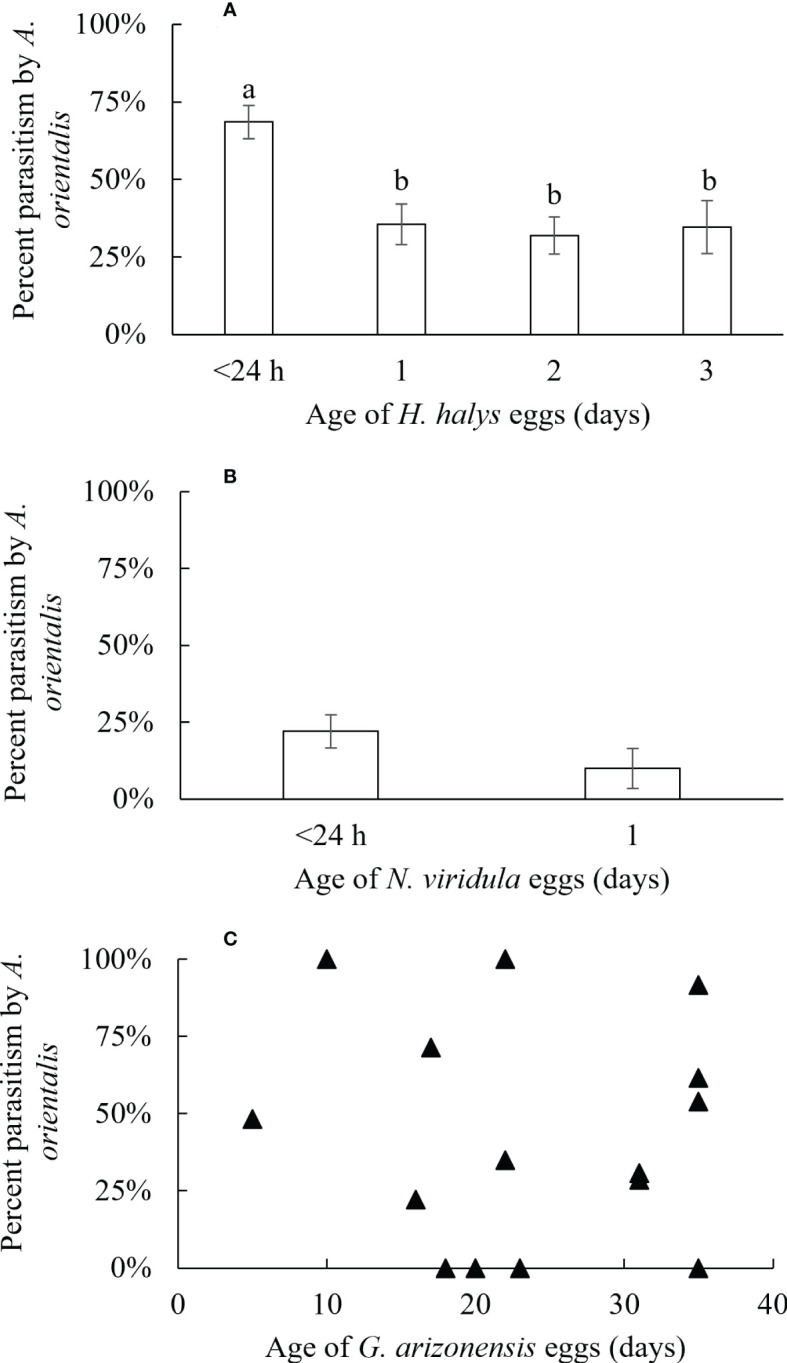
Percentage of parasitism (± SE) by *A orientalis* on non-target host eggs of **(A)** *H. halys* (<24 hours; n = 27, 1 day; n = 23; 2 days; n = 27, 3 days; n = 17), **(B)** *N. viridula* (<24 hours; n = 11, 1 day; n = 6) and **(C)** *G. arizonensis*. Different letters indicate significant differences for percent parasitism of eggs of different ages.

Male and female parasitoid offspring produced (female sex ratio) on each non-target species, number of repetitions per species (n), average number of eggs used per species per repetition and average parasitism rate of A. orientalis on eggs of each species.

(*) 100% of the A. orientalis females did not parasitize L. delicatula in the sequential exposure.

(**) target host.

a non-target host eggs of different ages. See section 1 and **Figure 2**.

A variation of this experiment that reduced female exposure time to non-target egg masses from five species [*C. hilaris*, *Gloveria arizonensis* Packard*, H. halys, N. viridula* and *Saturnia walterorum* Hogue & Johnson (**Table 1**)] was conducted. In this set of experiments, five *A. orientalis* females had a seven day pre-oviposition period with a male and access to a *L. delicatula* egg mass. Following this seven-day exposure period, individual mated females without males were exposed to non-target eggs (**Tables 1**, **2**) for 24 hours then moved to a target *L. delicatula* egg mass for 24 hours to confirm competency.

For both experimental designs, female *A. orientalis*, that did not produce offspring either on the non-target eggs or on the second exposure to the *L. delicatula* egg mass, were classified as “incompetent” and discarded from analyses. This rule was not followed for *Anisota oslari* Rothschild due to the high mortality (100%) of *A. orientalis* females following exposure to eggs of this species, and for *C. vittiger* and *C. vanduzeei*, due the low number of repetitions due to low egg availability (**Table 2**), which resulted from difficulty in acquiring sufficient test eggs of non-target species for experiments. Each *L. delicatula* egg mass (from pre-oviposition and post-non-target exposure) and non-target species eggs were held under the Beijing-fall regimen for one month, for development of parasitoid larvae. After this four-week period, eggs were transferred to 25°C, 16:8 L:D, and 75% R.H. for emergence of parasitoid offspring following previously published protocols (19). The host species from which *A. orientalis* emerged were recorded. Target eggs that did not produce parasitoids were dissected to detect failed parasitism (i.e., presence of dead parasitoid larvae or pupae were recorded). Percentage of parasitism was calculated as:


(1) $$\begin{array}{c} \%\;Parasitism\\ =\frac{Total\;number\;of\;parasitoids\;(i.e.,\;emerged\;adults\;+\;failed\;larvae\;+\;failed\;pupae)}{Total\;number\;of\;host\;eggs\;exposed}\text{x}\;100 \end{array}$$

Mortality of non-target host species due to exposure to *A. orientalis* was calculated using the Henderson–Tilton formula (Equation 2) (38), which calculates percent mortality based on the initial and final insect counts in the control relative to treatments with parasitoid exposure. Rates of naturally occurring mortality for non-target species host eggs were calculated with control eggs that were held under similar ambient conditions to test eggs but were not exposed to *A. orientalis*. The percentage of mortality by parasitoids was calculated as:


(2) $$\begin{array}{c} \%\;Mortality\;by\;parasitoids\\ =\left(1-\frac{\left(Average\;number\;of\;eggs\;in\;controls)\text{ x (}Number\;of\;juveniles\;after\;exposure\;to\;A.orientalis\right)}{\left(Number\;of\;eggs\;exposed)\text{ x (}Average\;number\;of\;juveniles\;in\;controls\right)}\right)\;\text{x}\;100 \end{array}$$

Using percent mortality caused by parasitoids and percent parasitism, percent mortality of non-target hosts resulting from parasitoid activity, but that did not result in *A. orientalis* offspring (i.e., excess mortality [due to host feeding and/or oviposition attempts]) was calculated with the equation:


(3) % Excess mortality =% Mortality by parasitoid−% Parasitism

Percent excess mortality caused by parasitoids to non-target hosts was compared with percent mortality in controls not exposed to parasitoids when the number of successfully completed repetitions for each treatment exceeded a minimum of three.

### 2.4 Choice host-testing

To assess host preference on parasitization by *A. orientalis* when given a simultaneous choice between eggs of non-target species and *L. delicatula*, choice host-tests were performed with two non-target species, *G. arizonensis* and *H. halys*. Pairs of egg masses of target and non-target species were presented simultaneously to female parasitoids ~48 hours of age in exposure arenas which were constructed using two stacked transparent U-shaped acrylic risers 15cm×15cm×15cm (SW Plastics F2191, Riverside, CA), that formed a rectangular cage 15cm×15cm×30cm with two open sides. One open face was covered with white semi-opaque no-see-um netting (Skeeta, Bradenton, FL) and the other was fitted with a 30cm-long sleeve sewn from no-see-um netting). Choice tests were run either for 24 hours or seven days. Inside arenas, egg masses were separated by 26 cm and randomly placed on the floor of arenas for each repetition. After exposure time, each group of eggs (target, non-target, and control eggs not exposed to parasitoids) were isolated in ventilated clear plastic test arenas (3 cm × 4 cm × 5 cm, see section 2) and held under the Beijing-fall regimen for four weeks before being moved to 25°C until parasitoids or immature non-target species emerged from eggs, or eggs were classified as dead and dissected for evidence of parasitism when possible.

### 2.5 *Anastatus orientalis* offspring sex ratio, fertility and size when reared from target and non-target hosts

*Anastatus orientalis* offspring that emerged from non-target host species and target host (i.e., *L. delicatula*) eggs were evaluated for offspring sex ratio, fertility of males and females, and adult size. Parasitoid sex ratio was calculated as the number of female parasitoids divided by the total number of female and male parasitoids combined that emerged from each experimental egg mass. Three different offspring fertility evaluations were performed on five non-target host species: *Actias luna* L., *Agapema anona* Ottolengui, *G. arizonensis*, *H. halys* and *P. fuliginosa.* First, males and females emerging from the same non-target host species (< 48 hours of age) were set up in test arenas (see section 2 for details). Second, males that emerged from non-target host species were coupled with unmated *A. orientalis* females that emerged from *L. delicatula* eggs. Finally, females that emerged from non-target host species were coupled with *A. orientalis* males that emerged from *L. delicatula* egg masses. All mating couples were exposed to *L. delicatula* egg masses for seven days and a thin smear of pure honey on the mesh of the ventilated lid of the test arena as a carbohydrate source. After seven days, male-female pairs were removed, and each egg mass was held under the Beijing-fall regimen for four weeks then moved to 25°C for the emergence of parasitoids and non-target hosts.

To evaluate the effect of host species on the size of *A. orientalis* male and female parasitoids that successfully emerged from non-target and target host eggs, measurements of right hind tibia lengths were used as a proxy for parasitoid size and subsequent fitness (i.e., parasitoids with larger hind tibia are assumed to be bigger and more fit than parasitoids with smaller tibia) (39, 40). Excised right hind tibiae were placed onto glass slides and covered with a second glass slide. Hind tibia length was measured from its point of attachment on the femur to the attachment point with the tarsi using a Leica S8AP0 microscope. Slide mounted hind tibiae were photographed at a magnification of 25 × with an attached Leica DMC2900 camera and length was measured using the Leica Application Suite version 4.6.2. A total of ~10 randomly selected *A. orientalis* males and females from each non-target host were measured and compared to 10 randomly selected males and females reared from *L. delicatula*.

To evaluate the effects of non-target host egg age on parasitism rates/host acceptance of *A. orientalis*, the age of eggs from all non-target host species exposed to *A. orientalis* was recorded. Data from eggs exposed to *A. orientalis* for seven days in the non-choice experiment (see section 2) were used. Three species, *G. arizonensis*, *N. viridula*, and *H. halys* resulted in sufficient repetitions and/or age variability to be analyzed. *Gloveria arizonensis* Packard eggs age ranged from 5 to 35 days old. *Nezara viridula* egg age used in this study were ≤24 hours of age. Finally, *H. halys* egg age ranged from <24 hours to three days of age. Percent parasitism was compared between egg age for each of these three species.

### 2.6 Statistical analysis

All statistical analyses were conducted in R version 4.1.3 (41) using the development environment RStudio (42). Percent excess mortality that resulted from non-reproductive behavior of *A. orientalis* was compared with natural-occurring mortality rates in the paired controls using a generalized linear model (GLM) with a quasi-binomial distribution to account for high variance in data sets. The relation between the hind tibia size of the male parasitoids offspring and the sex ratio of parasitoid offspring emerging from the same host was analyzed using linear regression. Differences in mean hind tibiae lengths between male and female parasitoids emerging from different non-target species eggs and target eggs were analyzed using ANOVA followed by a Tukey posthoc test at the 0.05 level of significance using the package ‘multcomp’. All means are presented ± standard error (SE).

## 7 Results

### 7.1 No-choice sequential host-testing experiments

In addition to the target host *L. delicatula*, eggs from a total of 34 non-target host species, however, eggs of three *Scaralina* spp. were pooled as *Scaralina* sp. giving a functional total of 32 species that were exposed to *A. orientalis* females in no-choice sequential host testing experiments. From the total of 244 female *A. orientalis* that completed the entire sequential exposure series (non-target and target), 23 (9.4%) females failed to parasitize the non-target host and the target host. They were considered incompetent and were excluded from data analyses. There were three exceptions for non-target hosts; *C. vittiger* and *C. vanduzeei*, due to the low number of repetitions because of the low numbers of non-target eggs available for testing, and *A. oslari* due to the high mortality of parasitoids (n =16; 100% of females tested died) after exposure to non-target eggs (**Table 2**). Additionally, 41 (16.8%) parasitoids were able to parasitize non-target host eggs and then failed to parasitize *L. delicatula* eggs. The results from these trials were included in data analyses.

*Anastatus orientalis* parasitized five species in the order Hemiptera: *A. thomasi* (Coreidae), *C. hilaris*, *H. halys* and *N. viridula* (all Pentatomidae) and *P. fuliginosa* (Fulgoridae) (**Table 2**). *Anastatus orientalis* parasitized 10 species in the order Lepidoptera: *A. luna, A. anona, Automeris cecrops pamina* Neumoegen*, Eupackardia calleta* Westwood, *Hyalophora euryalus* Boisduval*, Rothschildia cincta* Tepper*, S. walterorum* (Saturniidae), *G. arizonensis* (Lasiocampidae), *Pseudohemihyalea edwardsii* Packard (Erebidae) and *Pachysphinx occidentalis* Edwards (Sphingidae) (**Table 2**). The maximum percent parasitism of eggs for non-target hosts in Hemiptera and Lepidoptera were obtained on the native pentatomid, *C. hilaris*, and the native saturniid, *A. anona*, at 68.79% ± 12.41 and 76.48% ± 14.10, respectively.

**Figure 3:**
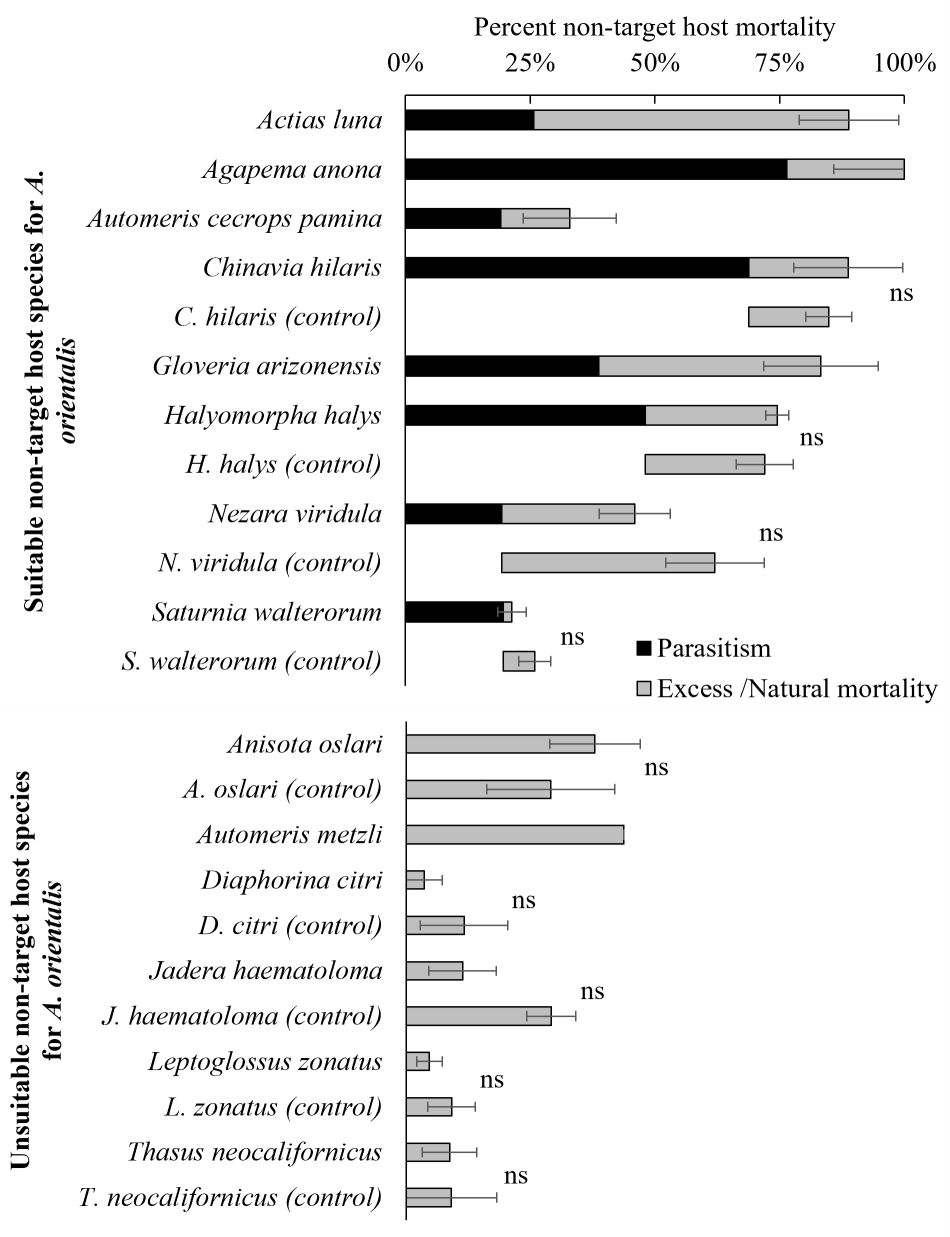
Percent non-target host mortality after exposure to *A. orientalis* from which parasitoids emerged (suitable hosts) and failed to emerge (unsuitable hosts). Species with no excess/natural mortality following parasitoid exposure were excluded from figure (Correction of mortality for exposed non-target host was null [Equation 2]). Total percent mortality of non-target hosts is the sum of the percentage of parasitism (in black) and the excess mortality (in grey) (± SE). (ns) Indicates no significant differences between the percent excess mortality after exposure to *A. orientalis* and the percentage of eggs alive without exposure to *A. orientalis* (control or naturally-occurring mortality).

Percent non-target host mortality of eggs exposed to *A. orientalis* was corrected with their paired controls (Equation 2) and excess mortality for non-target hosts following exposure to parasitoids was calculated (Equation 3). Excess mortality was compared across nine non-target hosts species (four suitable for parasitism and five unsuitable for parasitism, **Figure 3**) with their paired controls. Percent excess mortality resulting from death other than parasitism that resulted in the emergence of an adult parasitoid (i.e., mortality from host feeding and/or failed parasitism) for non-target host species were not affected by exposure to *A. orientalis* and no significant differences were found for nine species (*A. oslari*; *F*_1,18_ = 0.184, *P* = 0.67, *C. hilaris*; *F*_1,19_ = 0.11, *P* = 0.75, *D. citri*; *F*_1,4_ = 0.82, *P* = 0.41, *H. halys*; *F*_1,116_ = 0.097, *P* = 0.75, *J. haematoloma*; *F*_1,18_ = 3.5, *P* = 0.13, *L*. zonatus; F1,13 = 0.69, P *=* 0.42, *N. viridula*; *F*_1,30_ = 1.98, *P* = 0.16, *S. walterorum*; *F*_1,6_ = 0.71, *P* = 0.43, *T. neocalifornicus*; *F*_1,8_ = 4.89, *P* = 0.058) (**Figure 3**).

Of the five non-target host species exposed to *A. orientalis* females for 24 hours, three pentatomids, *C. hilaris*, *H. halys* and *N. viridula*, and two Lepidoptera species, *G. arizonensis* (Lasiocampidae) and *S. walterorum* (Saturniidae), were all parasitized by *A. orientalis*. The maximum average percent parasitism was observed for *S. walterorum* (42.96 ± 25.7) and the minimum average percent parasitism was recorded for *N. viridula* (4.79 ± 1.91) (**Table 2**).

The effect of non-target host egg age on parasitism by *A. orientalis* was evaluated for three species, *G. arizonensis*, *H. halys* and *N. viridula*. Rates of parasitism decreased as non-target host egg age increased for *H. halys* (*F*_1,92_ = 1.54, *P* < 0.001), and no effect of egg age on parasitism was observed for *N. viridula* (*F*_1,17_ = 1.54, *P* = 0.23). Similarly, increasing age of *G. arizonensis* eggs did not affect parasitism rates (*F*_1,13_ = 0.07, *P* = 0.79) (**Figure 2**).

### 7.2 Choice host-testing experiments

**Figure 4:**
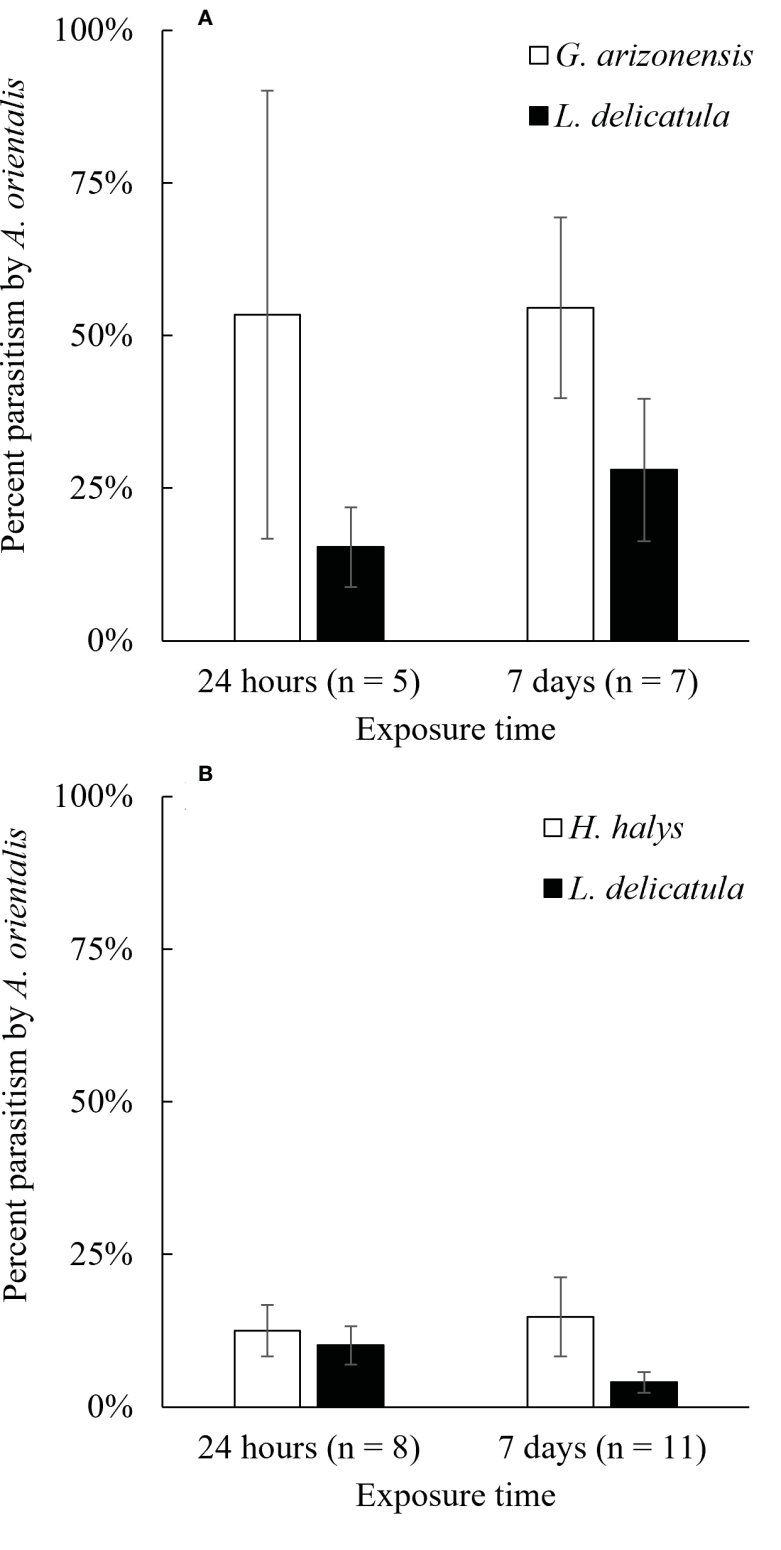
Average percent parasitism of non-target host eggs **(A)** *G. arizonensis* and **(B)** *H. halys*, when presented simultaneously with the target host, *L. delicatula* for two different exposure times, 24 hours and 7 days (n = number of repetitions).

The two non-target species used in choice experiments, *G. arizonensis* and *H. halys*, were parasitized in both exposure periods, 24 hours and 7 days, when exposed to *A. orientalis* in the presence of *L. delicatula* egg masses (**Figure 4**). For all the *G. arizonensis* vs *L. delicatula* choice trials (n = 12), four parasitoids failed to parasitize one of the two hosts species exposed; *G. arizonensis* and *L. delicatula* eggs were not parasitized three times and one time, respectively. For *H. halys* vs *L. delicatula* experiments (n = 19), three *A. orientalis* females did not parasitize either the target or the non-target host. Eight parasitoids failed to parasitize one of the two host species exposed; *H. halys* eggs were not parasitized in three trials and *L. delicatula* eggs were not parasitized in five trials.

### 7.3 *Anastatus orientalis* offspring from non-target hosts; sex ratio, male and female fertility, and size

#### 7.3.1 Sex ratio

A total of 15 non-target host species were suitable hosts for *A. orientalis* and a total of 1,870 parasitoids, 1,780 males and 90 females, emerged from susceptible non-target species following seven-day exposure time. The highest female sex ratio was obtained from *S. walterorum* eggs (0.5 ± 0.32) (there was one exception, *A. thomasi*, which only one egg was exposed and parasitized and resulted in a female parasitoid). Seven non-target species did not produce females after being parasitized by *A. orientalis* (i.e., parasitism of *A. anona*, *A. cecrops pamina*, *E. calleta*, *P. edwardsii*, *P. fuliginosa*, *P. occidentalis* and *R. cincta* eggs produced only male offspring) (**Table 2**).

The five non-target species exposed to *A. orientalis* for 24 hours resulted in a total of 137 parasitoids, 110 males, 24 females and three larvae. The highest sex ratio between these five species was obtained from *G. arizonensis* eggs (0.72 ± 0.21). Only *C. hilaris* and *H. halys* did not produce females (i.e., male offspring only produced) after being parasitized by *A. orientalis* (**Table 2**).

#### 7.3.2 Fertility

Male parasitoids were observed mating with females reared from the same non-target host species and female offspring were subsequently produced confirming mating was successful (*A. orientalis* is arrhenotokous and female offspring are produced from fertilized eggs). Consequently, male-female pairs of *A. orientalis* that emerged from the same non-target host species produced male and female offspring. (**Table 3**). Males that emerged from non-target host species successfully inseminated females emerging from *L. delicatula* egg masses as female offspring were produced from these male-female pairings (**Table 3**).

**Table 3:** Parasitism rates and offspring sex ratio (i.e., proportion of female offspring) produced by i) *A. orientalis* couples that emerged from five different non-target host species or ii) males that emerged from non-target host species mated with unmated females that emerged from *L. delicatula* eggs or iii) females that emerged from non-target host species mated with males that emerged from *L. delicatula* eggs (n = number of repetitions).

	Host source of *A. orientalis* mating pairs and resulting parasitism rates and offspring sex ratio											
	Male-female pair from same non-target host species				Male from non-target host, Female from *L. delicatula*				Female from non-target host, male from *L. delicatula*			
Non-target host species	Percentage of parasitism	(n)	sex ratio	(n)	Percentage of parasitism	(n)	sex ratio	(n)	Pecrentage of parasitism	(n)	sex ratio	(n)
*Actias luna*	10.39 ± 0.81	3	0.33 ± 0.19	3	58.19 ± 8.02	3	0.53 ± 0.26	3	–		–	
*Agapema anona*	–		–		11.64 ± 8.02	3	0.57	2	–		–	
*Gloveria arizonensis*	20.89 ± 13.27	7	0.88 ± 0.07	3	0	3	–		13.04*	1	–	
*Halyomorpha halys*	30.96 ± 7.93	13	0.36 ± 0.1	11	–		–		–		–	
*Poblicia fuliginosa*	–		–		0	1	–		–		–	

*Parasitoids failed to emerge and parasitism was calculated by counting numbers of parasitoid larvae (alive) in dissected eggs.

#### 7.3.3 Size

**Figure 5:**
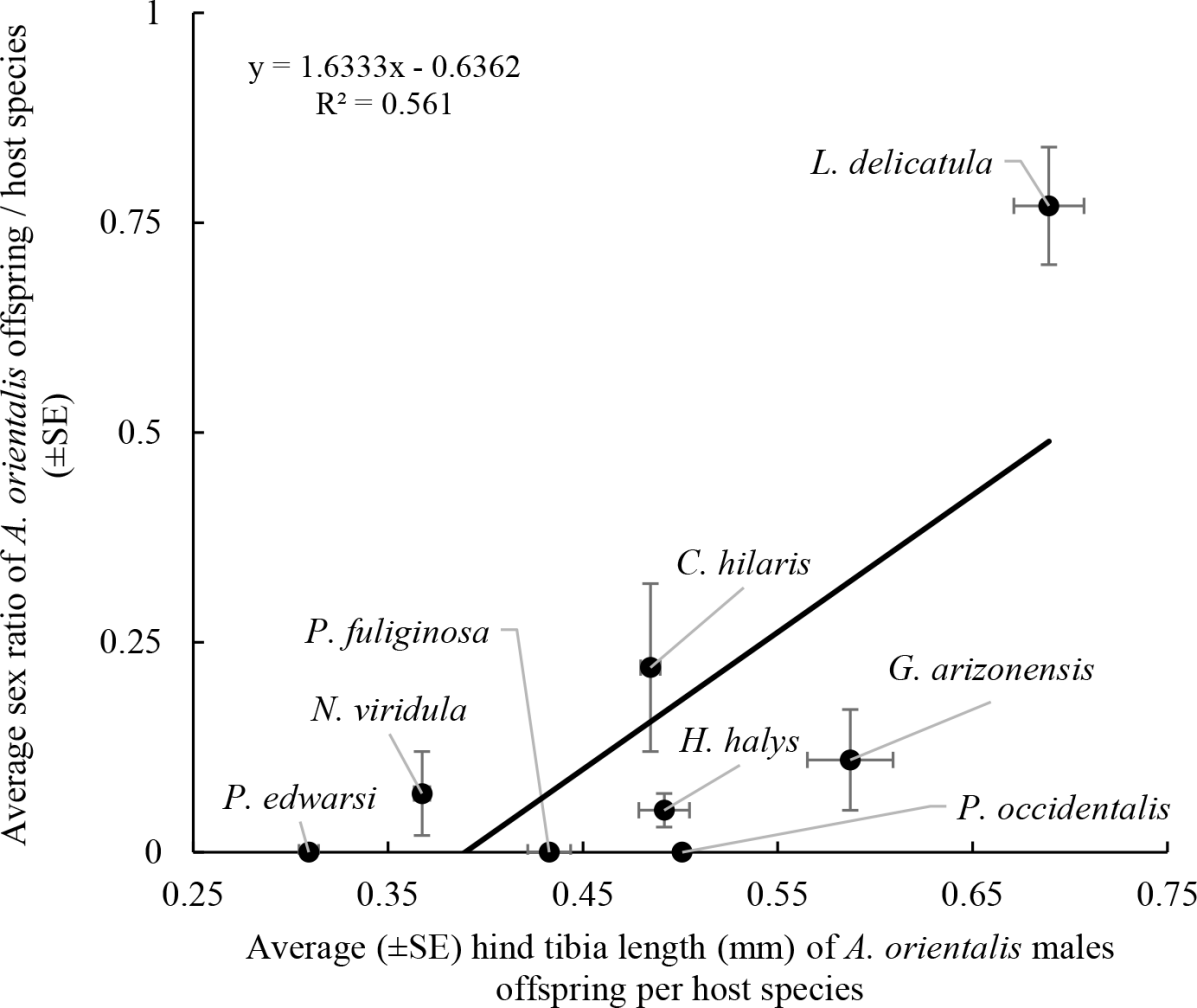
Relation between hind tibia length of *A. orientalis* offspring males emerging from each host (average values from **Table 4**) and sex ratio obtained from the same host (average values from **Table 2**).

A wide range of offspring sizes as well as a pronounced sexual dimorphism with larger females and smaller males was observed for *A. orientalis* when reared from non-target host species (**Table 4**). Average male hind tibia lengths ranged from 0.309 ± 0.005 mm (host: *P. edwardsii*) to 0.587 ± 0.022 mm (host: *G. arizonensis*). Average hind tibia lengths for females ranged from 0.613 ± 0.007 mm (host: *C. hilaris*) to 1.04 mm (host: *A. thomasi*) (**Table 4**). The largest *A. orientalis* males emerged from the target host, *L. delicatula* (*F*_6,62_ = 100.9, *P* < 0.001). The largest female emerged from the non-target host, *A. thomasi*. All other non-target hosts from which females emerged were smaller when compared with females that emerged from *L. delicatula* eggs (*F*_3,26_ = 285.4, *P* < 0.001) (**Table 4**). The average size of male offspring was significantly correlated with the sex ratio of the parasitoids emerging from the same host species (*F*_1,6_ = 7.668, *P* = 0.032; R^2^ = 0.561) (**Figure 5**). Non-target hosts that produced *A. orientalis* males were smaller and the sex ratio of emerged parasitoids was lower (i.e., male biased) and larger parasitoids typically emerged from host eggs that had female –biased sex ratios (**Figure 5**).

**Table 4:** Average hind tibia length (mm) of *A. orientalis* females and males that emerged from eggs of non-target species.

Host species	Average of hind tibia length (mm)			
	Males	n	Females	n
*Acanthocephala thomasi*	–	–	1.04	1
*Chinavia hilaris*	0.485 ± 0.005 cd	9	0.613 ± 0.007 c	4
*Gloveria arizonensis*	0.587 ± 0.022 b	10	0.881 ± 0.018 b	6
*Halyomorpha halys*	0.492 ± 0.013 c	10	0.629 ± 0.008 c	10
*Lycorma delicatula*	0.689 ± 0.018 a	10	0.959 ± 0.007 a	10
*Nezara viridula*	0.367 ± 0.004 e	10	–	–
*Pachysphinx occidentalis*	0.501	1	–	–
*Poblicia fuliginosa*	0.433 ± 0.011 d	10	–	–
*Pseudohemihyalea edwardsii*	0.309 ± 0.005 f	10	–	–

Different letters indicate significant differences between hind tibia size of parasitoids that emerged from different host species.

## 8 Discussion

This is the first study to assess the physiological host range and specificity of *A. orientalis* (Haplotype C) with respect to potential non-target species that occur in the southwestern U.S. In the quarantine laboratory, no-choice tests assessing the developmental suitability of non-target host species for *A. orientalis* demonstrated that 15 out of 34 non-target host species were suitable hosts for *A. orientalis*. This work indicated *H. halys* eggs could successfully support development of *A. orientalis* (offspring sex ratio was strongly male biased), a finding contrary to previous reports (43). *Antheraea* sp. (Saturniidae) eggs (i.e., *A. pernyi*) have been reported to be suitable reproductive hosts for *A. orientalis* (43). However, parasitization of *Antheraea oculea* Neumoegen eggs by *A. orientalis* was not recorded in this study. Five new hemipteran hosts in three families (Coreidae [*A. thomasi* {native}], Fulgoridae [*P. fuliginosa* {native}], and Pentatomidae [*C. hilaris* {native}, *H. halys* {invasive} *N. viridula* {invasive}]) and ten lepidopteran hosts in four families (Erebidae [*P. edwardsii* {native}], Lasiocampidae [*G. arizonensis* {native}], Saturniidae [*A. luna* {non-native}, *A. anona* {native}, *A. cecrops pamina* {native}, *E. calleta* {native}, *H. euryalus* {native}, *R. cincta* {native}, *S. walterorum* {native}] and Sphingidae [*P. occidentalis* {native}]) were identified as suitable hosts and are added to an increasing list of identified species that *A. orientalis* can successfully use as reproductive hosts in the laboratory (**Table 2**).

In terms of percent parasitism, eggs of two non-target species, *C. hilaris* (Pentatomidae [native]) and *A. anona* (Saturniidae [native]), were similar to or better hosts, than the target, *L. delicatula*. In the first case, *C. hilaris*, percent parasitism (68.79% ± 12.41; sex ratio 0.22 ± 0.1) was similar to the target, *L. delicatula* (58.24 ± 10.09; sex ratio 0.77 ± 0.07). This result may need to be interpreted cautiously, because *C. hilaris* eggs masses had on average a smaller number of eggs (17.73 ± 2.51) when compared to the average number of eggs per egg mass (39.08 ± 3.84) from the target, *L. delicatula*. In the second case, *A. anona*, percent parasitism was higher (76.48 ± 14.10; sex ratio 0) than the target *L. delicatula* (58.24 ± 10.09), and both egg masses were approximately equal in size (i.e., 42.2 ± 7.43 eggs and 39.08 ± 3.84 eggs for *A. anona* and *L. delicatula*, respectively). Collectively, data reported here and findings from companion studies (i.e., Broadley et al. [USDA], Submitted) suggest that *A. orientalis*, is at a minimum oligophagous, but more likely to be a polyphagous species.

Similar results from host specificity tests from other *Anastatus* spp. have been reported further supporting findings that *Anastatus* spp. potentially have broad host ranges. Stahl et al. (32, 33) studied the physiological host range of *A. bifasciatus* (this species is native to Europe) for use as a biological control agent against *H. halys*, an invasive pest in Europe. In this study, *A. bifasciatus* successfully parasitized eggs of eight pentatomid species (including *N. viridula* [tested in this study]) and 14 lepidopteran species belonging to seven different families (Endromidae, Erebidae, Lasiocampidae, Notodontidae, Papilionidae, Saturniidae, and Sphingidae). Results reported here indicate that *A. orientalis* can also parasitize hosts eggs from Erebidae, Lasiocampidae, Saturniidae and Sphingidae. Host range tests that expose *A. orientalis* females to eggs of Notodontidae and Papilionidae, two lepidopteran families with species representation in the southwest U.S., may be warranted to further understanding of potential non-target host use (the family Endromidae is not present in North America). For *Anastatus* spp., results reported here and those of Stahl et al. (32, 33), indicate strongly that selecting non-target species which are restricted to close taxonomic relatedness to the target pest (e.g., families in the Fulgoroidea [Hemiptera: Auchenorrhyncha]) may be inadequate as *Anastatus* spp. may tend to be generalists capable of parasitizing species across different orders.

No additional significant levels of excess mortality (i.e., mortality from causes other than parasitism) to non-target species exposed to *A. orientalis* was observed (**Figure 3**). Failure to detect excess mortality of non-target eggs exposed to *A. orientalis* when compared to levels of naturally-occurring mortality in control eggs not exposed to *A. orientalis* may have at least two explanations: i) parasitized non-target eggs develop successfully when parasitoid larvae and/or envenomation failed to kill the host egg (host eggs are incapable of encapsulating parasitoid eggs and larvae), or ii) *A. orientalis* females only host fed on eggs that were parasitized and it is possible that *A. orientalis* is a concurrent parasitoid (i.e., host feeds on parasitized eggs). Thus, egg mortality from host feeding alone was not observed and egg mortality was attributed to solely to parasitism. Additional studies confirming the lack of excess mortality due to unsuccessful parasitization or host feeding following exposure of non-target host species to *A. orientalis* are needed. Excess mortality of non-target host eggs following exposure to *A. orientalis* should be considered an important deleterious non-target impact if it occurs (44).

For three non-target host species tested, *A. oslari* (Saturniidae [native]), *C. vanduzeei* (Fulgoridae [native]) and *C. vittiger* (Coreidae [native]), all parasitoid females exposed to egg masses of these species failed to parasitize *L. delicatula* egg masses in sequential exposure tests. For *C. vanduzeei* and *C. vittiger*, too few non-target eggs were available for experiments and replication was low for each test species and therefore were not excluded from the results. Interestingly, for female *A. orientalis* exposed to *A. oslari* eggs excess egg mortality increased slightly but not significantly, and all females (n=16) died following exposure to *A. oslari* eggs prior to sequential exposure to *L. delicatula* egg masses. These results, a slight but non-significant increase in excess egg mortality and premature mortality of females, suggest that *A. orientalis* females may have host fed on *A. oslari* eggs and egg contents were possibly toxic to parasitoids. This could be explained by chemical protection of eggs by secondary plant compounds, like tannins, which are used as chemical defenses by host plants (i.e., *Quercus* spp.) of *A. oslari* (45). Sequestration of protective compounds, like tannins, in *A. oslari* eggs could reduce survivorship rates of third trophic level organisms like *A. orientalis* (46).

To evaluate a more realistic exposure time of *A. orientalis* to non-target species, 24 hour exposure tests (as opposed to a 7 day exposure period) were performed with five non-target species (*C. hilaris*, *G. arizonensis*, *H. halys*, *N. viridula* and *S. walterorum*). *Anastatus orientalis* was able to parasitize the five non-target species in this shorter exposure period following the seven-day pre-oviposition exposure period on *L. delicatula* eggs. These findings suggest that prior host exposure, especially to the target, *L. delicatula*, does not deter use of subsequent non-target host eggs. In addition, when non-target and target host eggs were exposed to *A. orientalis* females at the same time (i.e., choice experiments), that non-target species were attacked under both exposure time scenarios (i.e., 24 hours and 7 days). These results further suggest that *A. orientalis* is probably a generalist parasitoid capable of using any suitable non-target host species upon encounter. In many instances, female parasitoids engaged in parasitism within minutes of introduction into test arenas and contact with non-target eggs (pers. obs. F. Gomez Marco).

The age of non-target host eggs can affect the acceptance behavior of *A. orientalis* females and rates of successful parasitism. For example, percent parasitism by *A. orientalis* was higher on young eggs (<24 hours of age) vs. older eggs (>24 hours to 3 days) of *H. halys*. However, for two other non-target species, *G. arizonensis* and *N. viridula*, tested in egg age acceptance studies, no significant effect of egg age on parasitism was found. Therefore, age of non-target eggs and the non-target species may be an important covariables to consider when host tests are being designed and executed. Additionally, defensive chemical compounds (see above) may also affect the acceptance behavior of parasitoids (and survivorship rates) and this may also warrant consideration in design, analysis, and interpretation of host range tests (46).

The sex ratio of *A. orientalis* offspring was strongly dependent on host egg size. All non-target host eggs which were parasitized by *A. orientalis* with an average egg size visibly (not measured in this study) smaller than *L. delicatula* produced a male biased sex ratio (< 0.5) and smaller adult males and females. However, the sex ratio from hosts which produced smaller *A. orientalis* males decreased, indicating that there was a strong correlation between offspring size and offspring sex ratio. Only one non-target host with eggs larger than *L. delicatula* eggs (pers. obs. F. Gomez Marco) was used in our tests (*A. thomasi*) and this resulted in the largest female parasitoid (hind tibia size of 1.04 mm) obtained from host-range studies. These findings tentatively support conditional sex allocation theory, where, with decreasing host quality (i.e., host egg size in this study), parasitoid offspring sex ratio becomes more male biased and males are generally smaller, both of which correlate with decreased fitness (47). In support of results presented here, two previous studies, Hou et al. (48) and Stahl et al. (32), reported more male-biased sex ratios when *Anastatus* spp. were reared on host eggs that were smaller than the target host. Interestingly, differences in *A. orientalis* offspring sex ratio from two non-target host species, *C. hilaris* and *H. halys*, existed when egg masses were exposed for 24 hours (*C. hilaris* and *H. halys*: 0, no females) or seven days (*C. hilaris*: 0.22 ± 0.1, *H. halys*: 0.05 ± 0.02). This finding might indicate a preference of *A. orientalis* females to first oviposit (within at least the first 24 hours) non-fertilized eggs (i.e., produce male offspring) and then oviposit fertilized eggs (i.e., produce female offspring) when exposure times are longer and there is more time to repeatedly assess host quality. Sex ratio of offspring has important demographic implications as it affects rates of population grown. Male-biased progeny production on non-target hosts of marginal quality may limit or negate adverse population-level impacts on non-target species (32).

*Anastatus orientalis* offspring reared from non-target host species successfully parasitized *L. delicatula* egg masses. Percent parasitism and sex ratios resulting from mated couples that emerged from the same non-target host species were similar to values recorded for *A. orientalis* reared continuously on *L. delicatula*. Interestingly, males that emerged from *G. arizonensis* and *P. fuliginosa* when paired with unmated *A. orientalis* females that emerged from *L. delicatula* failed to produce any offspring. Due to the low number of repetitions for these experiments this finding should be viewed with caution. However, no obvious biological explanation (i.e., size of the males or inability to copulate with females) was observed to explain these results as unmated *A. orientalis* females should be able to oviposit unfertilized eggs that produce male offspring without mating with males that emerged from *G. arizonensis* and *P. fuliginosa*.

In laboratory studies in a quarantine facility, sequential no-choice and choice exposure studies that exposed female *A. orientalis* to non-target eggs of 34 native and non-native species in three orders and 12 families and target eggs (*L. delicatula*) for either 24 hours or 7 days, indicate that this egg parasitoid potentially has a wide host range as it successfully parasitized 15 species in 6 families in two orders (Hemiptera and Lepidoptera). Results presented here are for *A. orientalis* (Haplotype C). Other haplotypes of *A. orientalis* have been identified and are being assessed to determine if differences (i.e., greater specificity) in host preferences exist (20). A well-founded criticism of host-range tests is that parasitoids are constrained in small ventilated containers spaces with easily accessible hosts for long periods of time (i.e., 24 hours to 7 days) and are unable to engage in behaviors (e.g., rapid abandonment of patches with sub-optimal hosts) that could reduce or eliminate non-target use (49–52). Consequently, in the absence of comprehensive field data on host use and non-target species - target species - parasitoid phenology in the native range (i.e., China) from where *A. orientalis* was sourced, it is difficult to determine if high levels of non-target host use observed in host range tests reported here occurs in the field. Until detailed field data from the native range are available and based on results of host range tests reported here that suggest *A. orientalis* [and possibly most species of *Anastatus* (23–27, 32, 33)] has a very broad host range, use of this natural enemy in classical biological control of *L. delicatula* in the western U.S. should be assessed critically and with extreme caution.

## Data availability statement

The raw data supporting the conclusions of this article will be made available by the authors, without undue reservation.

## Author contributions

FG-M conceived and designed the experiments, performed collections and experiments, analyzed the data, prepared figures and/or tables, authored or reviewed drafts of the paper, and approved the final draft. DY performed collections, reviewed drafts of the paper, and approved the final draft. MR performed collections, reviewed drafts of the paper, and approved the final draft. MH obtained the funding, conceived and designed the experiments, performed collections authored or reviewed drafts of the paper, and approved the final draft. All authors contributed to the article and approved the submitted version.
